# The effect of a web-based training for improving primary health care providers’ knowledge about diabetes mellitus management in rural China: A pre-post intervention study

**DOI:** 10.1371/journal.pone.0222930

**Published:** 2019-09-24

**Authors:** Mu-Hong Wei, Xian-Zhen Chen, Xing-Xin Zhan, Zhi-Xia Zhang, Shao-Jing Yu, Wei-Rong Yan

**Affiliations:** 1 Department of Epidemiology and Biostatistics, School of Public Health, Tongji Medical College of Huazhong University of Science & Technology, Wuhan, China; 2 School of Nursing and Rehabilitation, Xinyu University, Xinyu, China; 3 Wuchang University of Technology, Wuhan, China; Mahidol University, THAILAND

## Abstract

**Background:**

The performance of primary health care providers regarding DM management is poor in rural China, and effective training methods for providers are urgently needed. This study aimed to evaluate the effect of web-based training for improving knowledge about DM management among primary health care providers in rural China and to further compare the effects of the training effect between primary health care providers with different backgrounds.

**Methods:**

A pre-post intervention study was conducted from April to August 2014. In this study, a total of 901 primary health care workers were recruited from six counties in Hubei province. To evaluate the effect of the web-based training, the knowledge achievement of participants was measured with multiple choice questions (MCQ) at baseline, at the end of two weeks of training and at three months after training. A mixed linear model (MLM) was used to measure group differences in the mean scores at baseline and follow-up.

**Results:**

After the web-based training, the knowledge scores of the village doctors increased from 73.58 at baseline to 89.98 at posttest and to 84.57 three months after the training. For township health workers, we observed an upward trend in scores from 78.87 at the pre-test to 91.72 at the second test, and at the three months after the training, the scores increased to 94.91. For village doctors, greater knowledge achievement was observed between the scores at baseline and after two weeks of training(adjusted difference: 3.55, P = 0.03) compared to that observed for the township health workers, while decreased their knowledge achievement between baseline and the third-test compared with that of township health workers (adjusted difference: 5.05, P = 0.01).

**Conclusions:**

This study suggested that web-based training was an effective method for improving the knowledge of primary health care providers about management of DM in remote areas. Compared with the effect of the training on village doctors, the training had a poor short-term effect on township health workers but a better long-term effect.

## Introduction

In recent years, the prevalence of diabetes mellitus (DM) has been steadily rising with the acceleration of globalization and urbanization, especially in low- and middle-income countries [1]. As the world’s largest developing economy, China has overtaken India as the center of the global diabetes epidemic [2]. The prevalence of DM in rural areas in China increased from 1.66% in 1995 to 5.31% in 2008 because of dietary shifts, lifestyle alterations and aging [3]. There is an urgent need for effective and efficient measures to improve DM management in rural China. In April 2009, the Chinese government launched a new health system reform to deliver basic public health services (BPHS) to all residents, and the health management of patients with DM is included in the BPHS plan [4]. Primary health care providers are the main force providing BPHS in rural China and are typically composed of village doctors and township health workers [5]. However, the current state of DM management in rural areas is not positive [6]. A nationally representative survey conducted in 2011 found that the performance of primary health care providers on DM management was poor and was related to their inadequate knowledge of DM prevention and control [4]. On-the-job training has been considered an effective way to improve the abilities of primary health care providers [7–9].

In recent years, the government has conducted numerous training programs, such as conference sessions, face-to-face instruction provided by senior doctors and remote/video education, to improve the competence of primary health care providers to deliver BPHS; among these training programs, face-to-face training is the most commonly used training method [10]. However, the disadvantages of face-to-face training, such as travel cost, inflexibility and limited training opportunities, resulted in it benefitting few trainees [11]. In rural China, most of the training for village doctors was held in township health centers or county health centers, which implied that village doctors needed to leave their posts to attend training [12]. However, there are only one or two doctors in most village clinics, and the clinics must be closed if the doctors need to join a training in another township or county [12, 13]. The travel time and cost for training cannot be ignored, especially in remote areas. Therefore, it is necessary to explore novel and effective training styles to complement the traditional face-to-face training format.

Along with the growth of the internet, web-based training has become a promising instructional method for reaching a large number of trainees [14]. It has been shown that web-based training methods have several advantages over traditional face-to-face training, such as its cost-effectiveness, ability to reach a wide range of audiences and unlimited training time and space [15, 16]. A meta-analysis by Cook DA et al reported that web-based training can be as effective as the traditional face-to-face training approach [17]. In low- and middle-income countries, such as Brazil, India, Egypt and South Africa, web-based training methods have been widely used to train clinical and nursing staff [15, 18, 19]. In China, there is a study reporting that web-based training successfully improved the knowledge of reproductive health topics among primary health care providers in rural areas of Yunnan province compared to the knowledge of providers receiving no intervention [20]. However, there are limited studies that have evaluated the effect of web-based training on improving knowledge about DM among primary health care providers in rural China.

Our previous qualitative study found that the majority of primary health care providers were willing to join web-based trainings, and most rural areas have already obtained internet access [12]. Therefore, it is feasible to carry out a web-based training program for primary health care providers in rural China.

This study was designed to explore the effect of web-based training for improving knowledge about DM management among primary health care providers. Considering the differences in educational backgrounds and job responsibilities between village doctors and township health workers, we further compared the effects of the training between village doctors and township health workers to provide a reference for future optimization of training contents and formats for different trainees.

## Materials and methods

This study had been approved by the Institutional Review Board of Tongji Medical College, Huazhong University of Science and Technology.

### Pilot study

Before starting this study, we conducted a pilot study between August and September 2013. Yiling district and Zhijiang city in Hubei province were selected as pilot areas using a convenient sampling method. A total of 86 primary health providers were included in the pilot study from village clinics and township health centers. The pilot study was used to test the contents of the training and provide feedback for the further modification of the contents. In the pilot study, the mean DM-knowledge scores before and after web-based training were also used to calculate the necessary sample size for this study.

### Design

This comparative, pre-post intervention study was used to evaluate the impact of web-based training. Study subjects were divided into a village-doctors group and a township-health-workers group to compare the short-term and long-term training effects on these two types of primary health care providers. Both groups received the same web-based training, and the DM-knowledge score was chosen as the main outcome variable to evaluate the effect of the web-based training.

### Study subjects and settings

The study was carried out in Ezhou, Xianning, and Yichang cities in Hubei province. Hubei province is located in central China, and its GDP (2737.922 billion) was ranked ninth among thirty-four provinces in China in 2014. The chosen cities were at different economic levels according to their GDPs (low: Ezhou; medium: Xianning; high: Yichang). Two counties were selected from each city according to their distance from the downtown area, close and remote. Finally, a total of 6 counties were included in the study (Zigui and Yiling from Yichang city, Chibi and Jiayu from Xianning city, and Echeng and liangzihu from Ezhou city).

The research subjects in our study were primary health care providers, either from the township health centers or village clinics of the selected counties. Primary health care providers who were responsible for the management and prevention of DM in local areas were included in the study. Primary health care providers were excluded if they could not access the internet or lacked basic computer skills. With the assistance of local CDC and health centers, research staff screened the basic information of primary health care workers. According to the inclusion and exclusion criteria, 950 qualified subjects were asked to provide written informed consent, while 49 refused to participate in this study. Finally, a total of 901 participants were recruited for this study. Only participants who had score data for the pretest and posttest were included in the final analysis.

### Intervention and data collection

Village doctors and township health workers received the same training contents, which focused on DM and included theoretical knowledge and case studies, via an online Moodle-based training platform from April to August 2014. The theoretical knowledge consisted of 4 parts: 1) the introduction of clinical features and complications of DM; 2) how to screen for complications among DM patients; 3) the treatment and management of patients with DM; and 4) health education for patients with DM. Each part was presented in the form of slides with synchronous voice interpretation. To consolidate the learning effect, several single-choice questions were added to the slides for each part. Four cases based on real-world events were delivered in the form of videos, which were recorded by senior experts in the prevention, diagnosis, treatment and management of patients with DM. The video was approximately 30 minutes in length for each case study. The training contents were designed primarily according to the requirements of the National Basic Public Health Service and actual local conditions, piloted in a small group of primary health care providers, and later modified multiple times based on feedback from DM experts and local health administrators specializing in DM management.

Participants in both the village-doctors group and the township-health-workers group were given 2 weeks to complete the web-based training (1 week for the theoretical knowledge section and 1 week for the case studies). Before starting the training, all the participants were given one week to familiarize themselves with the training platform. An instruction manual with detailed instructions on the use of the online training platform was provided to all trainees. During the training period, two discussion forums were established separately for the village-doctors group and the township-health-workers group, and only the participants in the same group could ask and discuss questions with each other in their discussion forum. Two facilitators (members from the research group) were designated to solve the problems encountered by the trainees in the learning process, such as failure to log on to the training platform and inability to understand the learning contents in each forum. A four-level supervision group consisting of members of the research group, the city CDC, the county CDC and the township health centers was established to monitor and supervise trainees’ online learning during the two-week training period. The trainees’ learning time could be tracked on the platform. If they did not complete the online course in time, the supervisors would remind them by phone to complete the training.

Knowledge achievement was assessed by a 10-item questionnaire with multiple choice questions (MCQ). The questionnaire was designed based on the training contents and then reviewed by experts. Then, we conducted a pilot study among 86 participants from Yiling district and Zhijiang city and found that the Cronbach’s alpha coefficient was 0.76. In the questionnaire, each question was assigned a value of 1, and the final score was calculated as the percentage of correct answers (total score was 100%). A total of three tests were given to measure the participants’ knowledge of DM at different times in our study. Only after completing the pretest could the participants access the training contents. Once they finished the two weeks of training, participants were asked to take a posttest via the internet; then, at 3 months after finishing the training, all subjects were asked to take a third test, which was used to evaluate the long-term training effect. The three tests contained the same questions but in different orders. During the study period, the training platform was open to all participants, and participants were able to access the training contents on the platform even though the training was over.

### Sample size

The sample size was calculated with PASS v11.0. The information used in the sample size calculation, such as the baseline score (72.45), knowledge score change (5.74) and the standard deviation (16.85) of the mean difference among primary health care providers before training, was acquired from the pilot study. When the 2-sided significance level was set at 5% and the study power was set at 90%, 145 subjects were needed to detect a pre-post difference among the primary health care providers, based on the information from the pilot study. Considering that thirty percent of the subjects could be lost to follow-up during the study, 44 subjects were added to each group (189 subjects). The proportion of village doctors and township health workers in the sample was set at 2:1 reflecting the actual ratio between the two groups in each town. Therefore, at least 189 township health workers and 378 village doctors were included in the study.

### Statistical analysis

The qualitative variables, such as sex, educational level and major of the participants, were described as numbers and percentages, and a chi-square test was used to compare the characteristics of the village doctors and township health workers. For the quantitative variables, t-tests were used to test group differences for normally distributed data, and Wilcoxon rank-sum tests were used for non-normally distributed data. A mixed linear model (MLM) was used to measure group differences in the mean scores at baseline and follow-up, with effects of assessment time, group, and group×time and covariates of age, sex, educational level and major.

All statistical analyses were implemented in SAS version 9.1 (SAS Institute). The P values reported in the study were 2-tailed, and P<0.05 was considered statistically significant.

## Results

**Fig 1:**
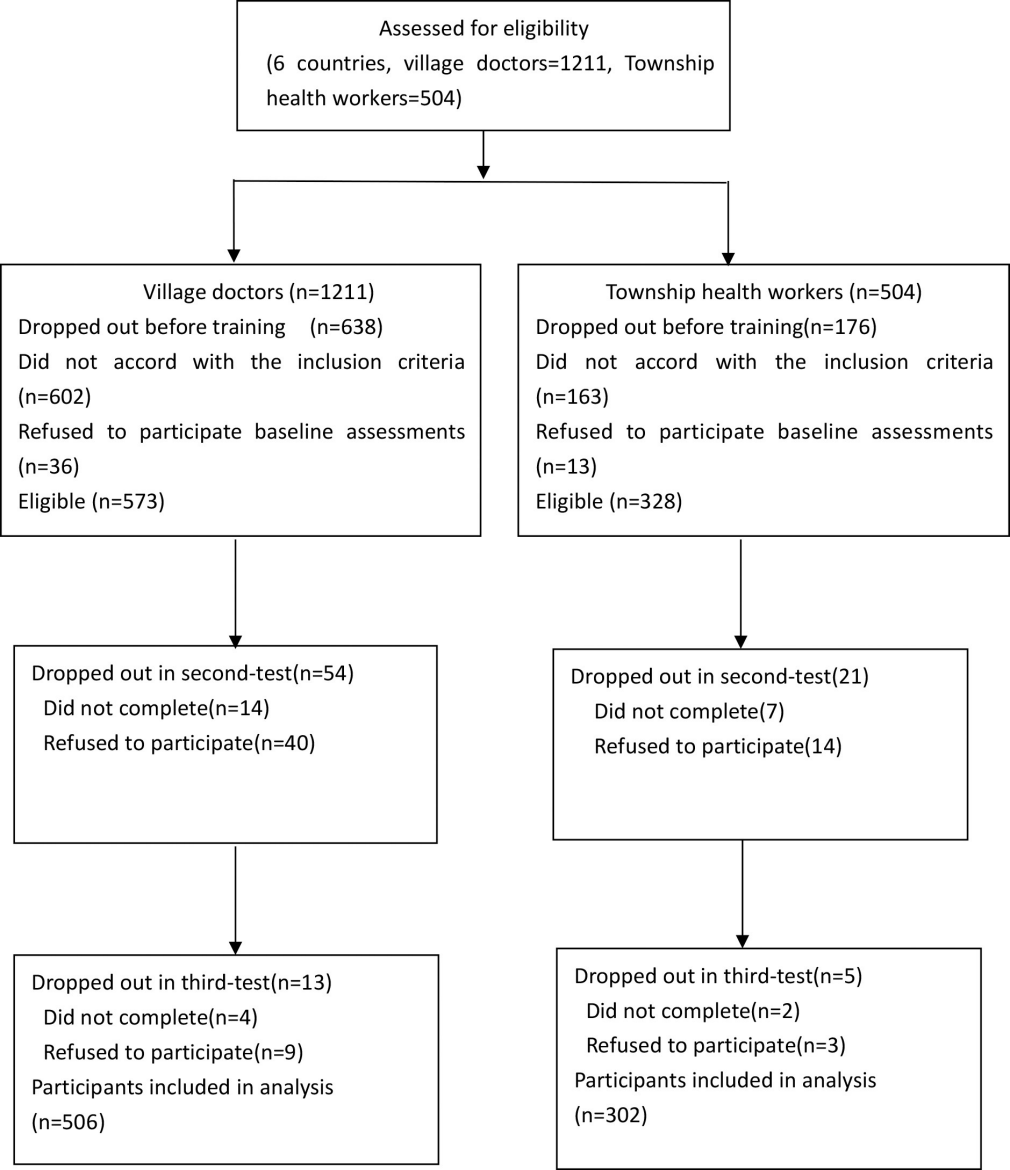
Research flow chart.

Fig 1 shows the flow of participants through the study. A total of 901 individuals were eligible for recruitment, including 573 village doctors and 328 township health workers. Due to dropouts and failure to complete the examinations, 506 village doctors and 302 township health workers were included in the final analysis. Among the participants excluded in the final analysis, 18 participants were dropped for lack of both the second and third test-score data and 75 for lack of the second test-score data.

Table 1 describes the characteristics of the study subjects. The mean age of township health workers was younger than that of village doctors (p<0.001). Approximately 25.65% of village doctors were over 50 years old. The proportion of males in the village-doctors group (73.47%) was much higher than that in the township-health-workers group (29.88%) (p<0.001). Compared to village doctors, township health workers had better educational backgrounds (p<0.001).

**Table 1:** Demographic characteristics of the participants.

Variable	Village doctors(n = 573)	township health workers (n = 328)	χ2/Z	p
age, n(%)				
≤30	33(5.76)	73(22.26)	81.33	<0.001
31–40	192(33.51)	117(35.67)		
41–50	201(35.08)	111(33.84)		
≥51	147(25.65)	27(8.23)		
Median(P_25_,P_75_)	43(37,51)	39(32,43)	-8.19	<0.001
sex, n(%)				
Male	421(73.47)	98(29.88)	162.33	<0.001
Female	152(26.53)	230(70.12)		
educational level, n(%)				
middle school or below^a^	33(5.76)	2(0.61)	181.74	<0.001
high school/vocational school	482(84.12)	163(49.7)		
junior college or above	58(10.12)	163(49.7)		
Specialty, n(%)				
clinical medicine^b^	421(73.47)	112(34.15)	235.85	<0.001
Nursing	42(7.33)	140(42.68)		
preventive medicine	37(6.46)	29(8.84)		
traditional Chinese medicine	54(9.42)	4(1.22)		
other^c^	19(3.32)	43(13.11)		
Years of Working				
<5	143(24.96)	162(49.39)	61.41	<0.001
5-	235(41.01)	108(32.93)		
10-	79(13.79)	29(8.84)		
≥20	116(20.24)	29(8.84)		

middle school or below

^a^: primary school, middle school

clinical medicine

^b^: Western medicine, Chinese traditional and Western medicine

other

^c^: refers to stomatology, pharmacological medicine, imageology

Table 2 describes the baseline knowledge score, assessed before the training. A significant difference was detected between the two groups at baseline (p<0.001), which suggested that township health workers had better knowledge of than village doctors.

**Table 2:** Difference in the baseline knowledge score between the two group.

Group	N	Pretest (%)	t	p
Village doctors	506	73.58	3.35	<0.001
Township health workers	302	78.87		

**Fig 2:**
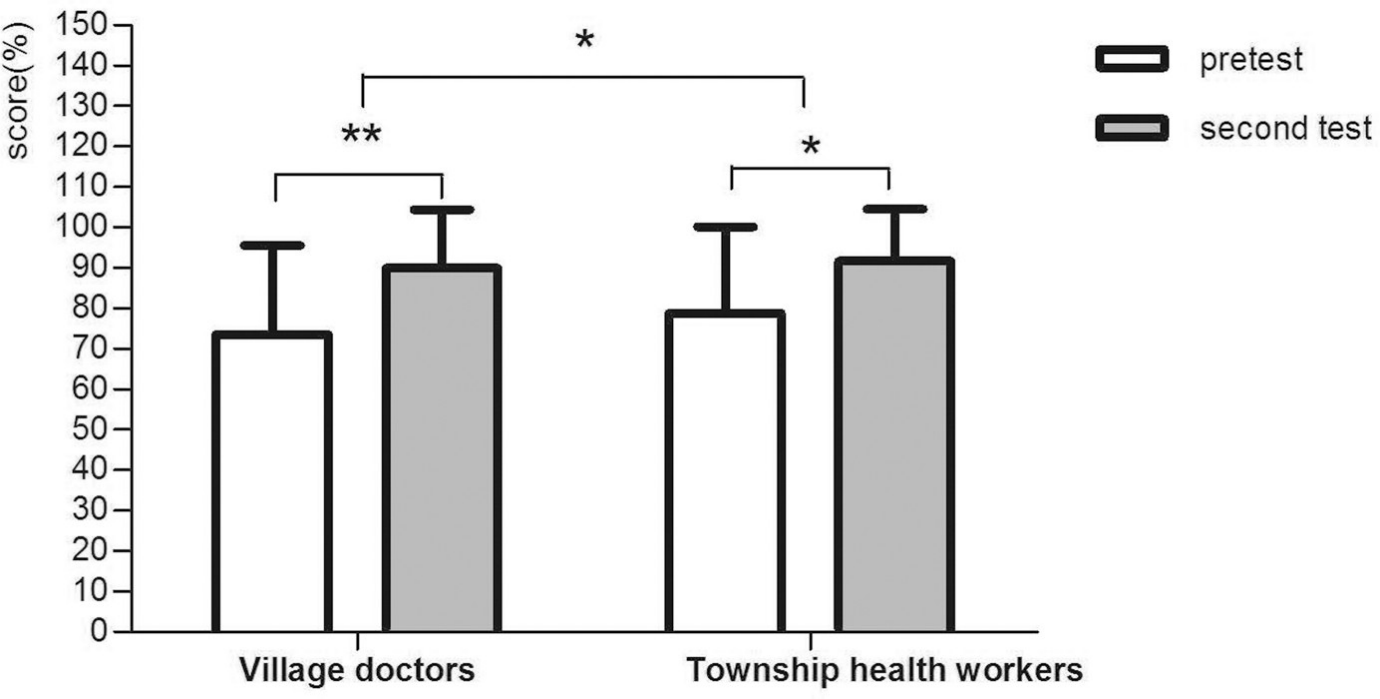
The short-term training effect. *0.01≤P<0.05, **P<0.01. Adjusted for age, sex, educational level and major.

Fig 2 presents the short-term effect of the training. After the training, both village doctors and township heath workers had improved knowledge (p<0.001 and p = 0.01). Compared to township health workers, village doctors had acquired more knowledge with an adjusted difference of 3.55 (p = 0.03).

**Fig 3:**
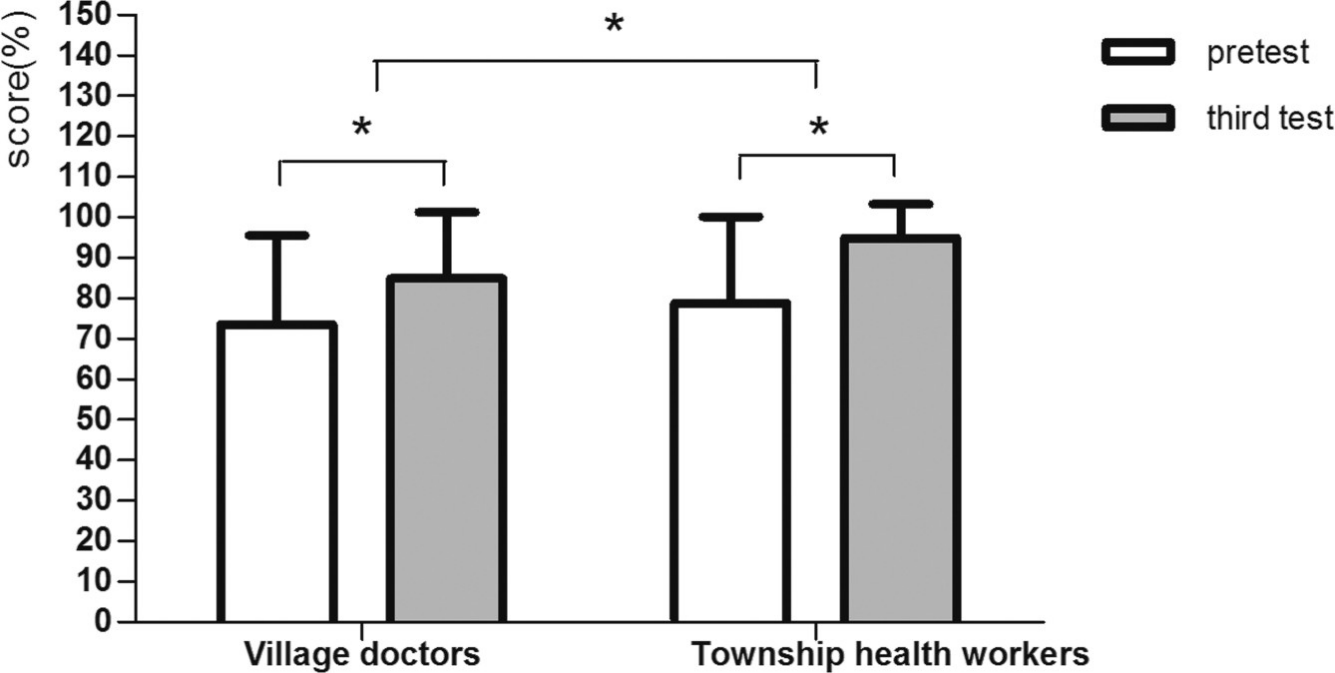
The long-term training effect. *0.01≤P<0.05, **P<0.01. Adjusted for age, sex, educational level and major.

Fig 3 shows the long-term effect of the training. Three months after the training, the township health workers had a better knowledge of DM compared with that of the village doctors. The adjusted difference between the two groups was 5.05 (p = 0.01). Both were higher than those at baseline (10.99 and 16.04, respectively).

## Discussion

We found that web-based training was an effective method for improving primary health care providers’ knowledge about DM in rural China. The findings also showed that township health workers had better baseline knowledge scores than those of village doctors. Considering the difference in baseline scores, a mixed linear model was used to compare the changes between the pretest and posttest scores between the two groups. The results suggested that the training had a better short-term effect on the village doctors, but it had a better long-term effect on the township health workers.

After the web-based training, the knowledge scores of both the village doctors and township health providers increased considerably, which suggested that web-based training can effectively improve primary health providers’ knowledge about DM. This is supported by a meta-analysis of 201 studies that found that web-based training was associated with large positive effects compared with no intervention [17]. A pre-post intervention study conducted among rural physicians in the USA showed that the use of web-based training could increase knowledge about DM among physicians, which was consistent with our findings [21]. Similarly, Tang et al. reported that web-based training effectively increased reproductive health knowledge among health providers in rural China [20]. The results of the previous studies were consistent with ours, which suggested that web-based training can effectively improve the health-related knowledge of rural health professionals.

The current study showed that township health workers had better knowledge about DM at baseline than village doctors. As the main force providing BPHS, township health workers hold the responsibility of supervising and managing the BPHS work of village doctors [7, 22]. Considering the job responsibility of township health workers, it is not surprising that township health workers had a better baseline knowledge about DM than village doctors. Another possible explanation may be the difference in their educational background. Before working at township health centers, township health workers had at least 3 years of medical education and had to pass the Licensed Assistant Doctors Examination organized by the Department of Health. However, the vast majority of village doctors graduated from a technical secondary school [23]. The difference in educational background between the two groups was also found in our results.

In the process of learning, academic performance will rise rapidly at an early stage and slow down, or even become stagnant, in the late stage; this is called the plateau phenomenon in education psychology [24]. The plateau phenomenon is related to multiple factors, one of which is that the difficulty of learning increases gradually [25]. Progress can be made only when more difficult and complex knowledge is mastered, which takes more time and energy with progressive learning. This is further supported by our results showing that village doctors with less baseline knowledge made greater progress in the short term compared with those who had more knowledge at baseline. Compared with village doctors, township health workers were in the plateau period of learning and required more time and energy to make progress.

If a training strategy works, the effect should be long-lasting. A three-month follow-up was used to assess the long-term effect of the web-based training used in this study. During the 3 months following the training, primary health providers could still log on to the platform to access the previous training contents but without supervision and guidance.

The long-term training effect was always positively related with persistent motivation [26]. On the one hand, township health workers have the responsibilities of supervising and administering all the BPHS work of village doctors [7]. The demands of the job may encourage township health workers to continue to take part in training seriously to deepen and strengthen their knowledge. However, many village doctors have not realized the importance of BPHS jobs. A study by Ding et al indicated that village doctors in some areas were not willing to provide BPHS for a long time because of the low reimbursement payments, which may partly affect the enthusiasm for continuous participation in the training [27, 28]. Village doctors not only provide BPHS but also need to provide medical service to rural residents, which may give them less free time to attend training due to their heavy workloads [27]. Considering these reasons, it is not surprising that there was a better long-term training effect among township health workers.

There are several limitations of our study that should be acknowledged. First, we selected only six counties from three cities with different economic levels in Hubei province, which may limit the generalizability of the findings to other areas. Second, the study was only designed to explore the change in knowledge about DM and did not measure the change of practices. Future research should focus on whether increased knowledge can be converted to better health practices. Third, a pre-post comparative design was used in our study to analyze the effect of web-based training. Therefore, we need to interpret observed changes to our intervention with caution due to the lack of a control group.

### Conclusions

Our study evaluated the effect of a web-based training on improving the knowledge of DM regarding, which indicated that web-based training was a feasible training method in remote areas. However, the training effect was different between primary health care providers with different backgrounds. Therefore, future research is needed to identify the factors influencing the training effect to provide guidance for developing effective training programs for primary health providers from different backgrounds.

## Supporting information

S1 Appendix Questionnaires. (DOCX)

S1 File Data-set. (XLS)
