# Supplementary material for: The effect of a web-based training for improving primary health care providers’ knowledge about diabetes mellitus management in rural China: A pre-post intervention study
Source: PLoS One. 2019 Sep 24;14(9):e0222930. doi: 10.1371/journal.pone.0222930 (PMC6759173; doi:10.1371/journal.pone.0222930)
Supplement: S1 Appendix — (DOCX) [file pone.0222930.s001.docx]

Part 1: Basic information

1. Name____ Sex_____ Age____

2. Department: _____

3. The length of time for health services:______ (years)

4. The educational background: _____

(1) primary school or below (2) middle school (3) high school/vocational school (4) junior college (5) university or above

5. The major: ____

(1) clinical medicine (2) nursing (3) stomatology (4) pharmacological medicine (5) preventive medicine (6) traditional Chinese medicine (7) Chinese traditional and Western medicine (8) imageology (9) other__

Part 2: Basic knowledge

1. The biggest obstacle that affects the control of diabetic blood sugar is:()
2. Hypoglycemia  B. Early insulin use C. Excessive weight D. Insulin resistance
3. The best time to measure fasting blood glucose is: ()

A. 6 to 8 a.m. B. 6 to 10 a.m. C. 8 to 10 a.m. D. 8 to 12 a.m.

3. In the Chinese guidelines for the prevention and treatment of type 2 diabetes, the ideal fasting glucose is:()

A. <5.0 mmol/L B. <6.1 mmol/L C. <6.0 mmol/L D. <7.0 mmol/L

4. The correct view on blood glucose monitoring in patients with diabetes is:( )

A. When fasting and postprandial blood glucose levels are normal, there is no need to monitor HbA1c

B. Fasting blood glucose is more important than postprandial blood glucose

C. Postprandial blood glucose is more important than fasting blood glucose

D. Patients with diabetes should monitor both immediate blood glucose (fasting and postprandial) and long-term control levels (HbA1c).

5. Which is incorrect regarding the role of exercise therapy in lowering blood sugar in diabetic patients:( )

A. Exercise promotes the use of muscle glycogen and glucose in the blood

B. Exercise increases insulin sensitivity

C. Exercise can reduce the occurrence of diabetes complications

D. The more exercise, the better the blood sugar lowering effect

6. The prime time for diabetics to exercise is:( )

A. Morning exercise B. 1 hour after meal C. One hour before dinner

D. Before sleep

7. An incorrect understanding of diabetic blood glucose standards is: ( )

A. Early compliance, early benefits

B. Achieve full blood glucose standards

C. Fine hypoglycemic, safe standard

D. It does not matter if you are following them, as long as you are on treatment

8. In the Chinese guidelines for the prevention and treatment of type 2 diabetes, the gold standard for blood glucose is: ( )

1. Fasting blood-glucose B. Postprandial blood sugar C. HbA1c D. Blood Pressure

9. The five arms of diabetes treatment include: ( )

A.Diet and exercise therapy

B.Massage therapy

C.Drug therapy

D. Tobacco and alcohol

E. Education and psychotherapy, self-monitoring

10. The following is true about the risk factors for diabetes: ( )

A. Obesity is associated with type 2 diabetes

B. A healthy diet can not only help diabetics reach the blood sugar target but also help healthy people avoid diabetes

C. To overcome all kinds of incorrect psychological barriers to diabetes, blood sugar should be treated as early as possible to achieve the target levels

D. There was no significant association between reduced exercise and diabetes

第一部分 个人基本情况

1.姓名___________ 性别_________ 年龄__________

2.您目前所在的单位：___________________________

3.您参加基本公共卫生服务工作： 年

4.您的学历： ① 小学 ② 初中 ③ 高中/职高/中专 ④ 大专 ⑤ 本科及以上

5.您所学的专业：① 临床 ② 护理 ③ 药学 ④ 口腔 ⑤ 中医 ⑥ 预防医学 ⑦ 中西医 ⑧影像 ⑨其他_______

第二部分 基础知识

1.影响糖尿病的血糖控制达标的最大障碍是：（）

A 低血糖的发生 B 早期使用胰岛素

C 体重过度增加 D 胰岛素抵抗

2.测空腹血糖的最佳时间是：（）

A 早上6至8时 B 早上6至10时

C 早上8至10时 D 早上8至12时

3.在中国的2型糖尿病防治指南中，理想的空腹血糖是：（）

A ＜5.0 mmol/L B ＜6.1 mmol/L

C ＜6.0 mmol/L D ＜7.0 mmol/L

4.关于糖尿病患者的血糖监测正确的观点是：（）

A空腹血糖和餐后血糖正常了，就没必要监测糖化血红蛋白

B 空腹血糖比餐后血糖重要

C 餐后血糖比空腹血糖重要

D 糖尿病患者即要监测即时血糖（空腹和餐后），又要兼顾长期的控制水平（糖化血红蛋白）

5.关于糖尿病患者运动疗法降低血糖的作用错误的是：（）

A 运动能促进肌糖原和血液中葡萄糖的利用

B 运动能增加胰岛素的敏感性

C 可减少糖尿病并发症的发生

D 运动量越大，降低血糖效果越好

6.糖尿病患者运动的黄金时段是：（）

A 晨起锻炼 B 餐后1小时

C 餐前1 小时 D 睡前

7.对糖尿病血糖达标认识错误的是：（）

A 早达标、早获益 B 实现血糖全面达标

C 精细降糖，安全达标 D 只要坚持治疗，达不达标不重要

8.在中国2型糖尿病防治指南中，血糖达标的金标准是：（）

A 空腹血糖 B 餐后血糖

C 糖化血红蛋白 D 血压

9.糖尿病治疗的五驾马车包括：（）

A饮食控制和运动疗法 B按摩理疗

C药物治疗 D戒烟戒酒 E教育及心理治疗、自我监测

10.以下关于糖尿病的发病因素正确的说法是：（）

A 肥胖与2型糖尿病的发病相关

B 健康饮食方式不仅可以帮助糖尿病患者血糖达标，还有助于健康人群远离糖尿病

C 克服各种错误的糖尿病心理障碍，有助于血糖尽早达标

D 运动减少与糖尿病发病没有明显关联
